# Does the economic growth target overweight induce more polluting activities? Evidence from China

**DOI:** 10.1371/journal.pone.0282675

**Published:** 2023-03-07

**Authors:** ZhengZheng Shi, Hongwen Chen, Kunxian Chen

**Affiliations:** 1 School of Economics, Xiamen University, Xiamen, China; 2 School of Tourism, Nanchang University, Nanchang, China; 3 School of Economics, Jinan University, Guangzhou, China; Hainan University, CHINA

## Abstract

In China, official promotion evaluation based on economic performance motivates local governments to develop high economic growth targets, which has played an active role in boosting China’s economic growth in the past decades, whereas its environmental consequences have not been fully exploited. This paper finds that the economic growth target overweight has a stronger positive impact on the output of high-polluting industries than on the output of low-polluting industries, thus inducing more polluting activities. To deal with the issues of reverse causality and omitted variables bias, we take an instrumental variable approach. Examining mechanisms, we show that economic growth target overweight promotes polluting activities through the deregulation of the polluting activities in high-polluting industries. We also find an increase in the impact of the economic growth target overweight after the 2008 global economic crisis. Our study provides new evidence for explaining the dual presence of rapid economic growth and heavy environmental pollution in China.

## 1. Introduction

Environmental damage is one of the biggest threats to human well-being in the short and long run and has been an eye-catching research topic in economics and environmental science [1–3]. One type of environmental damage is the climate warming. It is documented that carbon dioxide (CO2), one of the leading greenhouse gases, has risen to its highest level in the past 800,000 years [4], and will continue to grow [5]. Massive carbon emission is considered the main cause of climate warming, which is associated with many diseases such as mental disorders [6], primary hypertension [7], and diabetes [8]. Ambient pollution is another type of environmental damage, which has adverse effects on humans in many aspects, including health and life expectancy [9, 10], productive [11, 12], and short-run cognition [13]. Therefore, it is urgent to take action to curb the deteriorating environmental issue, but before it we have to have a good knowledge of the causes of it.

One possible cause is the economic growth, as reflected in the empirical correlation between economic growth and environmental pollution, the so-called environmental Kuznets curve (EKC). The idea behind the EKC is that an inverted U relationship exists between ambient levels of pollution and GDP per capita [14]. The rising part of the EKC is what is going on now in China. Since the launch of the reform and opening up in 1978, China’s economic performance has been remarkable. For instance, according to the data from the National Bureau of Statistics of China, its GDP grew more than 200 times from 1978 to 2018 (at 1978 constant prices), a 9.4% average annual growth rate, which is much higher than the world’s average during the same period. However, rapid economic growth is also accompanied by serious environmental damage [15, 16]. According to the data from the Ministry of Ecology and Environment, China’s economic losses due to environmental damage stood at about 2 trillion yuan in 2015. Clearly, environmental pollution has been a significant impediment to China’s long-term economic development.

Chinese economy is intertwined with its political system. One of salient features of the China’s political economy is that local officials’ chances of promotion are bound up with the economic growth in their jurisdictions. In general, the higher economic growth rate is, the higher promotion chances the local officials get [17, 18]. This incentive mechanism encourages local officials to pay much attention to GDP growth to compete for promotions, the so called “promotion tournament” [19], which is a crucial contributor to China’s economic success in the past decades [18]. For more promotion chances, lower-level government typically set higher growth targets than upper-level ones (i.e., the “top-down amplification”) [20], generating the economic growth targets overweight (EGTO).

**Fig 1:**
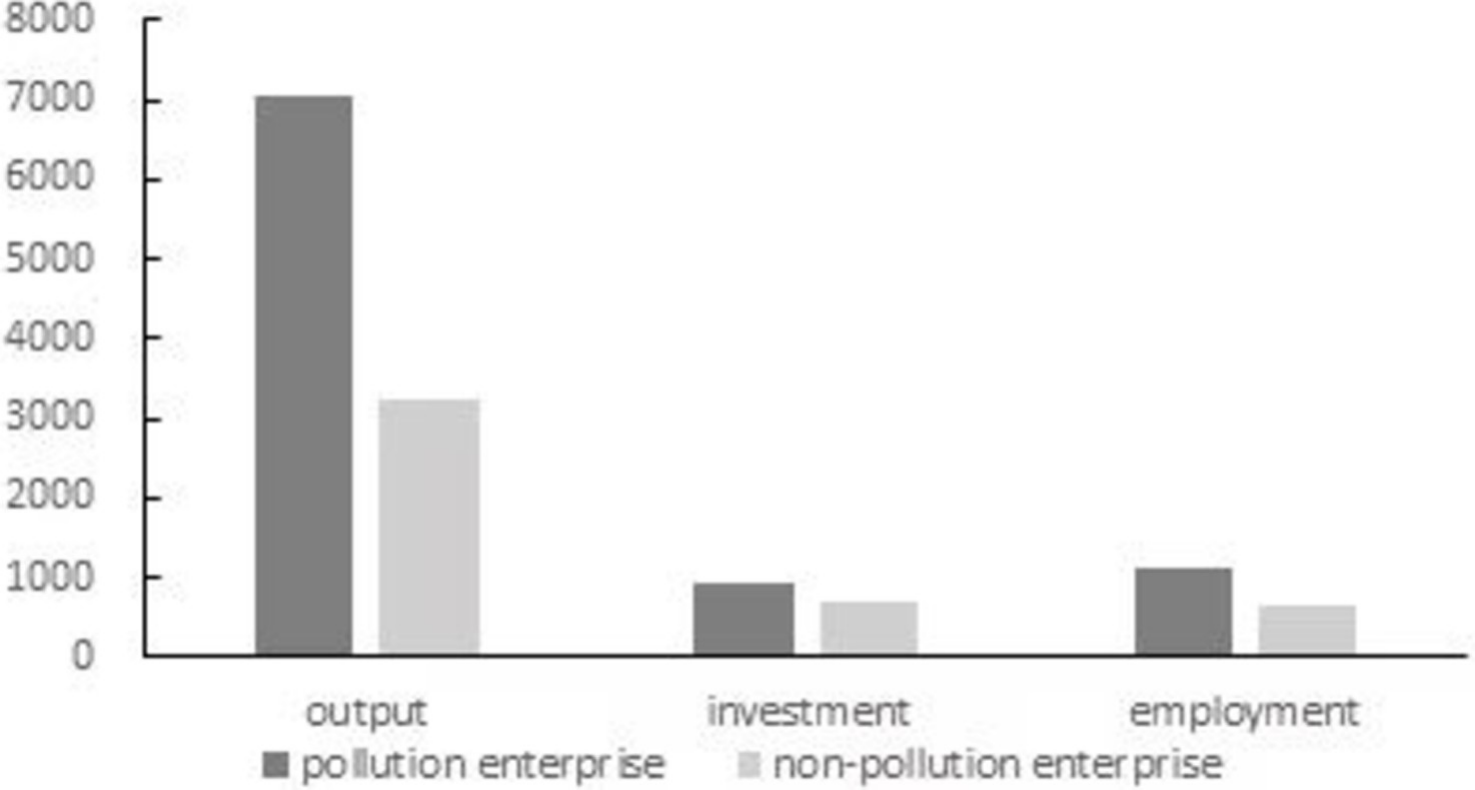
Average output, investment, and employment of enterprises of high-polluting and low-polluting industries. **Note:** The unit of output and investment is 10000 yuan, and the unit of employment is 100 people. We calculate the average output, employment, and investment of high-polluting and low-polluting enterprises based on the enterprise data in the ASIF.

EGTO brings great pressure of boosting the economy to the local officials, which may lead to the neglection of local governments to environmental protection. As shown in Fig 1, the average output, investment, and employment of the pollution companies are all higher than those of the non-pollution companies. This implies that local officials have incentives to take more care of pollution companies so as to achieve the growth targets [21], which, however, may exert negative effect on environment quality. This argument is evidenced by many studies. For example, it is found that local officials often neglect pollution controls when developing the economy, thus resulting in environmental degradation [22]. Some research shows that government officials have incentives to support local firms through tolerating their heavy pollution and even protecting them from being penalized for producing excessive pollution [23]. A research finds that higher economic growth target can lead to lower intensity of the environmental regulation [20]. Finally, there is a collusion between governments and enterprises, which has been a severe problem hindering environmental protection and green development in China [24, 25]. With the discussion above, a natural question is that how does EGTO affect the environmental pollution? This has not been fully exploited by previous research and is the focus of our paper.

This paper aims to explore the impact of the EGTO on polluting activities in China. Using data from the Annual Survey of Industrial Firms (ASIF) and the annual work reports of provincial and prefecture-level governments, we find that the economic growth target overweight can induce more pollution activities, which is reflected in the stronger positive impact of EGTO on the output of high-polluting industries than the output of low-polluting industries. Moreover, the effect of EGTO on pollution activities varies across the ownership and scale of enterprises and the release date of the economic growth target. We provide evidence that EGTO increases polluting activities through reducing the environmental regulations of polluting activities of high-polluting industries. We further explore the influence of the global economic crisis in 2008, and document an increase in the effect of EGTO after the crisis.

Existing literature on environmental pollution has deepened our understanding of pollution from the perspective of economic and institutional factors. For example, some scholars have paid attention to economic growth and reform [26–28], trade and foreign investment [29–31], industrial structure and agglomeration [32, 33]. Some scholars also focus on market segmentation [34], fiscal decentralization [35], and political turnover [36]. This paper investigates whether and how municipal governments’ economic growth targets influence pollution activities. The setting of an economic growth target involves both economic and non-economic factors. Exploring the impact of EGTO can provide a new explanation for the relationship between China’s economic growth and environmental pollution activities. Cities pursuing higher economic growth not only attract the transfer of pollution but also encourage the expansion of pollution. The pollution transfer caused by environmental regulation has attracted the attention of many scholars [15, 37, 38]. Our research finds that the EGTO also causes the transfer of pollution. In the process of China’s development, the economically developed regions set higher targets before the first decade of the 20th century, but after that, the underdeveloped regions set higher targets, which may cause the poor people in the underdeveloped areas to suffer from environmental damage. This issue should be paid close attention to.

In addition, existing studies attribute the success of China’s economy to its unique political system, which effectively encourages economic competition among officials and helps the central government to ensure that prefectural governments are consistent with national policy objectives [39, 40]. We supplement this traditional view. Just like the central government’s economic growth accounting for local governments, due to the principal-agent relationship between them, there may be inconsistent goals.

The rest of the paper is structured as follows. Section 2 introduces the background of economic growth target, promotion system, and environmental regulation in China. Section 3 presents the empirical strategy. Section 4 introduces the data and variables used in our analysis. Section 5 shows the empirical results, including baseline regression results, robustness checks, and heterogeneity analysis. Section 6 examines the mechanism through which EGTO affect polluting activities. Section 7 investigates the time variation of the impact of EGTO on polluting activities before and after the 2008 global financial crisis. Finally, Section 8 concludes and makes some policy suggestions.

## 2. Institution background

### 2.1 The relationship between economic growth target and official promotion

In China, the central government assigns economic growth rate targets to provincial governments, and the provincial governments redistribute the targets to subordinate prefecture-level governments. In order to supervise and inspire local officials, the central government adopts the cadre evaluation system to cascade financial and personnel incentives to local governments along the hierarchy [41]. Based on the performance evaluation system, local officials in China give priority to the policy formulated by higher authorities [42]. There are assessment objectives in policy areas such as economic growth and production safety, and officials go all out to achieve these goals. In terms of economic growth, local governments not only passively accept the tasks of their superiors but actively set higher targets to impress their superiors. For example, most provinces are usually expected to have higher growth than the central government. Prefecture-level cities also set higher growth targets than the provincial targets, resulting in the “layer upon layer”. The career prospects of officials are closely related to the degree to which the targets are fulfilled [41, 43, 44]. Local officials who achieve their goals have more promotion opportunities and will receive more financial transfers from higher-level governments, while those who fail to achieve their goals are punished, such as dismissal and demotion [43, 45]. Since 1978, economic growth has become a top priority for governments at all levels. The promotion tournament on economic growth has been a common phenomenon in municipal and provincial governments [44, 45]. Some scholars [46] verify that economic performance has a significantly positive impact on the promotion opportunities of local officials. The promotion incentive urges local officials to set higher economic goals. One research [43] demonstrates that the target of the higher-level government is an important reference for the subordinate government when setting its own target. The chance of promotion depends on relative performance, i.e., local officials need to outperform their peers, which motivates local governments to set higher economic growth targets [19]. However, the economic growth tournament also makes local governments myopic in economic efficiency. For example, public expenditures are of low profits but play an important role in increasing GDP will be favored by governments [42, 43].

### 2.2 Pollution control practice of the Chinese government

In response to the increasingly severe environmental damage, the two-control zone (TCZ) was proposed in 1998 to reduce acid rain and sulfur dioxide pollution. Starting from the 10th Five-Year Plan in 2001, each five-year plan has set targets for pollutant emission reduction. The main leaders of the cities in areas that fail to achieve the target or have the most serious pollution each year will be interviewed by the Ministry of Environmental Protection (MEP). In 2003, President Hu Jintao formally put forward the Scientific Outlook on Development (SOD) to seek the coordinated development of economic, environmental, and social issues. In order to address the serious water pollution problem in China, hundreds of water monitoring stations have been installed along the main rivers in China, and the importance of water quality monitoring has been emphasized. The MEP set clear water quality targets for monitoring stations and systematically published the readings, which significantly curbed the discharge of pollutants [47]. In 2008, environmental factors were added to the promotion evaluation of officials. This year, MEP was upgraded to the Ministry of Ecological Environment (MEE). The improvement of the administrative level means the increase of the regulatory power of environmental protection departments. However, in the absence of transparency in air quality data, local officials can whitewash environmental quality data to cope with the assessment [48], which still cannot effectively encourage local officials to carry out substantive environmental governance [23, 49].

In short, promotion pressure makes officials focus more on economic growth than environmental protection. This study aims to prove the causality between economic growth target overweight of the prefecture-level governments and pollution activities, the possible mechanism, and the change of this causality under the pressure of economic and environmental protection in different periods.

## 3. Model specification

Since higher EGTO requires more efforts in developing the economy and high-polluting industries can bring more economic benefits compared with low-polluting industries, we conjecture that the impact of EGTO on the output of high-polluting industries is larger than the output of low-pollution industries, and set the following interaction model to identify the heterogeneous effect:

$$y_{ict}=\beta_{0}+\beta_{1}EGTO_{ct}*dirty_{i}+\varphi_{it}+\tau_{ic}+\lambda_{ct}+\epsilon_{ict}$$ (1)

where *y*_*ict*_ is the output of industry i of city c in year t. *EGTO*_*ct*_ denotes the economic growth target overweight of city c in year t, which is measured by the economic growth target gap between city c and subordinated province. *dirty_i_* is an indicator variable that equals one for high-polluting industries, and zero for low-polluting industries. *φ*_*it*_ are industry-year fixed effect, which captures all time-variant and time-invariant industry characteristics, such as industry-specific technologies and industrial policies.; *τ*_*ic*_ are industry-city fixed effects that absort different cities to have differential levels of industrial agglomeration and industrial policies. *λ*_*ct*_ are city-year fixed effects which controls all time-variant and time-invariant city characteristics, such as geographical location, productivity change, economic development level, public policy, official characteristics and turnover, etc. The coefficient of interest is *β*_1_, which captures the heterogeneous effect of EGTO on high-polluting industries and low-polluting industries. In specific, if *β*_1_ > 0, the EGTO has a larger impact on the output of high-polluting industries.

## 4. Data and variables

Our estimation sample is a panel of 260 Chinese cities in 23 provinces from 2006 to 2014. We collect economic growth targets data from official government websites and documents. Other data on city-level variables are from the China City Statistical Yearbook. All nominal values are adjusted by the industrial producer price index (PPI), which is from the National Bureau of Statistics of China, to the 2006 price level.

### 4.1 Measuring the intensity of industrial pollution

We use the pollution intensity index (PII) to measure the intensity of industrial polluting activities following a research [50], which is defined as:

$${\text{pII}}_{ij}=(W_{ij}/Q_{i})/(\sum_{i=1}^{39}W_{ij}/\sum_{i=1}^{39}Q_{i})$$ (2)

where i and j index industries and pollutants, respectively. Due to the data availability, we focus on three types of pollutants. These are: wastewater, smoke, and sulfur dioxide. *W*_*ij*_ denotes the emission of pollutant j of industry i. *Q*_*i*_ represents the value added of industry i. The PII of wastewater, smoke, and sulfur dioxide for each industry are shown in S1 Appendix. We refer to industries with PII>1 as “high-polluting” industries, and industries with PII<1 as “low-polluting” industries. S2 Appendix displays the list of high-polluting industries for different pollutants. Pollution emission and value-added data of each industry are from China Statistical Yearbook and China Industrial Statistical Yearbook, respectively.

### 4.2 Industry-level data

The output and investment data of industries are from the Annual Survey of Industrial Firms (ASIF, National Bureau of Statistics 2006–2014). The database contains non-state-owned enterprises with an output value of more than 5 million and all state-owned enterprises. These total output values account for more than 95% of the national industrial output value, which is a representative sample. Based on firm-level data, we calculated the annual gross output value of each two-digit industry in each city from 2006 to 2014. As the pollution industries are determined by the double-digit classification method according to the existing literature, they are divided into high-polluting industries and low-polluting industries based on the pollution emission intensity. The dependent variable is the logarithm of the total output value of high-polluting and low-polluting industries, which measures the intensity of polluting activities. Table 1 displays the definition and summary statistics for the main variables used in our analysis.

**Table 1:** Definition and summary statistics for the main variables used in our analysis.

Variable	Definition	Obs	Mean	Min	Max
EGTO	Target gap between city and province (%)	2170	2.08	−7.10	15
EGTO2	Target gap between city and the central government (%)	2170	2.51	-5.7	18
Target decision-making time before province		2170	0.41	0	1
GDP per capita (Log)		2170	10.21	4.62	13.06
Population density	People per square kilometer	2170	493.79	22.31	2641.17
Envir_r	Average environment-related text proportion (%)	2170	2.01	0.58	7.44
Output value of polluting industry (100 million)		2170	485.65	4.43	3931.33
Output value of air-polluting industry (100 million)		2170	429.73	3.71	3518.07
Output value of water-polluting industry (100 million)		2170	391.05	3.69	3448.16

## 5. Empirical results

### 5.1. Main results

Table 2 reports the estimation results of model (1). Columns 1 and 2 show that the coefficient of the interaction term between EGTO and the high-polluting industry dummy is positive and statistically significant for air-polluting industries both with and without controlling for fixed effects, which demonstrates that EGTO has a stronger positive impact on the output of high air-pollution industries. Thus, EGTO can induce more air-polluting activities. Columns 3 and 4 display the effect of EGTO on water-polluting activities. The coefficient of the interaction is lower but remains positive and significant, which suggests that EGTO can facilitate water-polluting activities. Finally, we focus on industries that feature both high air pollution and water pollution. Columns 5 and 6 report the results. The coefficients of the interactions are still significantly positive. Taken together, the results in Table 2 suggest that EGTO will induce more polluting activities, thus having a negative effect on the environment.

**Table 2:** Effect of EGTO on polluting activities.

	Dependent variables: industrial output					
	Air-polluting industry		Water-polluting industry		Both	
	(1)	(2)	(3)	(4)	(5)	(6)
EGTO *dirty	0.083***	0.065***	0.058***	0.045***	0.074***	0.061***
	(0.023)	(0.017)	(0.018)	(0.016)	(0.018)	(0.017)
Industry-year FE	Y	Y	Y	Y	Y	Y
City-year FE	N	Y	N	Y	N	Y
City-industry FE	N	Y	N	Y	N	Y
Observations	4340	4340	4340	4340	4340	4340
R-squared	0.867	0.924	0.728	0.836	0.843	0.889

Notes: Standard errors are clustered at the industry level.

*** p<0.01,

** p<0.05,

* p<0.1.

### 5.2 Robustness checks

#### 5.2.1 Alternative measures of high-polluting industry and EGTO

We now check the robustness of our baseline estimates. We first examine the sensitivity of the baseline estimates to the use of alternative measures of high-polluting industries and EGTO. In this paper, we use the pollution intensity index to measure high-polluting and low-polluting industries. In this subsection, we follow the criterion of the First Nationwide Pollution Source Survey (FNPSS), regard the key pollution source industries as the high-polluting industries, and others as low-polluting industries. The new high-polluting industry dummy is labeled as *dirty*2. Column 1 in Table 3 presents the estimates. The coefficient of the interaction remains positive and significant.

**Table 3:** Alternative measures of high-polluting industry and EGTO.

	Dependent variables: industrial output			
	Both	Air-polluting industry	Water-polluting industry	Both
	(1)	(2)	(3)	(4)
EGTO *dirty2	0.061***			
	(0.017)			
EGTO2 *dirty		0.095***	0.089***	0.090***
		(0.032)	(0.031)	(0.030)
Industry-year FE	Y	Y	Y	Y
City-year FE	Y	Y	Y	Y
City-industrial FE	Y	Y	Y	Y
Observations	4340	4340	4340	4340
R-squared	0.910	0.937	0.895	0.906

Notes: Standard errors are clustered at the industry level.

*** p<0.01,

** p<0.05,

* p<0.1.

Next, we check the robustness to using an alternative measure of the EGTO. Since the political competition of officials may occur between cities in different provinces, we use the difference between the growth targets of prefecture-level cities and the central government to measure the overweight of economic target, and denote it as EGTO2. Results are shown in Columns 2–4 in Table 3. The coefficients for the interaction terms remain positive and significant in all specifications.

#### 5.2.2 Endogeneity

Although we have controlled for a large set of fixed effects in Eq (1), we still suffer from the omitted variables bias. For example, government officials in cities with different levels of economic development and population density may attach differential importance to pollution. Specifically, people in developed and densely populated cities generally place more weight on environmental quality. Thus, GDP per capita and population density are two potential omitted variables that confound the estimates. To correct the bias, we further control for the interaction between GDP per capita and the high-polluting industry dummy, and the interaction between annual population density and the high-polluting industry dummy. Since the economic growth target is usually set at the beginning of a year, we lag GDP per capita and population density by one year. Results are presented in panel A of Table 4. Columns 1–3 show that we obtain qualitatively identical results when we condition on the two controls—i.e., the coefficients of the interaction terms between EGTO and the high-polluting industry indicator are all positive and statistically significant. In addition, there are fewer polluting activities in cities with high GDP per capita and high population density.

**Table 4:** Baseline results after addressing endogeneity problems.

	Dependent variables: industrial output		
	Air-polluting industry	Water-polluting industry	Both
	(1)	(2)	(3)
***Panel A*. *Controlling for GDP per capita and population density***			
EGTO *dirty	0.065***	0.042**	0.054***
	(0.020)	(0.020)	(0.019)
Log GDP per capita (t-1)*dirty	-0.134**	-0.186***	-0.175**
	(0.072)	(0.062)	(0.070)
Population density (t-1)*dirty	-0.179***	-0.102**	-0.151***
	(0.058)	(0.049)	(0.056)
Industry-year FE	Y	Y	Y
City-year FE	Y	Y	Y
City-industry FE	Y	Y	Y
Observations	4340	4340	4340
R-squared	0.942	0.901	0.920
***Panel B*. *IV estimates***			
EGTO *dirty	0.405***	0.328***	0.357***
	(0.138)	(0.117)	(0.120)
Industry-year FE	Y	Y	Y
City-year FE	Y	Y	Y
KP F-statistic	103.15	61.47	83.79
Observations	4340	4340	4340
R-squared	0.834	0.791	0.816

Notes: Standard errors are clustered at the industry level.

*** p<0.01,

** p<0.05,

* p<0.1. The KP F-statistic is the Kleibergen-Paap Wald rk F-statistic for weak identification in the first stage.

Although having controlled for a set of fixed effects and the two aforementioned covariates, we can’t completely address omitted variables bias through this way because there are unobservable variables affecting both EGTO and polluting activities. Besides omitted variables bias, another type of endogeneity is the reverse causality. That is, the relative output of high-polluting industries may shape the government’s decision of setting economic growth target, thus affecting EGTO. To address both problems, we employ an instrumental-variables (IV) strategy using the number of prefecture-level cities in a province as the instrument for EGTO.

Causal inference of the IV strategy requires that the selected instrument satisfy both the exclusion restriction and relevance condition, i.e., the number of prefecture-level cities in a province influences polluting activities only through EGTO. We now elucidate the validity of our instrument based on the two criteria. First, in China, local officials are promoted by higher-level officials, but the quota for promotion is a scarce resource. In general, the more prefecture-level cities a province comprises, the more intense competition between these cities of the province is. The more competitive environment for promotion encourages local officials to set higher economic growth targets and thus leads to larger EGTO. Second, arguably the number of prefecture-level cities in a province is mainly determined by historical and geographic factors (e.g., conditional on area, geographically more flat provinces generally have more subordinate cities due to the ease of construction and transportation) rather than unobserved drivers of polluting activities. Hence it satisfies the exclusion restriction. Since the number of prefecture-level cities in a province does not change over time, and thus the interaction term between EGTO and the high-polluting industry indicator is subsumed into city-industry fixed effects, we do not control city-industry fixed effects when regressing using the instrument.

Panel B in Table 7 reports the IV estimates. In all three columns, the coefficients of the interactions are still positive and statistically significant. The Kleibergen-Paap Wald rk F-statistic (KP) for weak identification is much larger than the Stock-Yogo critical value of 10. Note that IV estimates are about four times as large as their OLS counterparts, which suggests that OLS estimates underestimate the effect of EGTO.

#### 5.2.3 Transfer of polluting activity

If local governments set high economic growth target, they will try hard to attract investments so that the number of new enterprises increase while the number of old enterprises remains steady. To detect the heterogenous effects, we use the information from the enterprise’s opening year to calculate the number of new and old enterprises in each industry of a city. Specifically, old enterprises are those whose opening year is 2005 or earlier; otherwise, they are new enterprises.

Columns 1–3 in Table 5 show that EGTO has a stronger positive impact on the number of new enterprises in high-polluting industries. In contrast, we don’t see significant effects on old enterprises from columns 4–6, which indicates that EGTO increases pollution activities through attracting new enterprises to high-polluting industries.

**Table 5:** Effect of EGTO on the number of new and old enterprises.

Dependent variables:	Log (number of new firms +1)			Log (number of old firms +1)		
	Air- polluting industry	Water- polluting industry	Both	Air- polluting industry	Water- polluting industry	Both
	(1)	(2)	(3)	(4)	(5)	(6)
EGTO *dirty	0.048***	0.037**	0.042**	0.027	0.010	0.022
	(0.016)	(0.019)	(0.020)	(0.019)	(0.021)	(0.018)
Industry-year FE	Y	Y	Y	Y	Y	Y
City-year FE	Y	Y	Y	Y	Y	Y
City-industry FE	Y	Y	Y	Y	Y	Y
Observations	4340	4340	4340	4340	4340	4340
R-squared	0.924	0.871	0.915	0.857	0.823	0.846

Notes: Standard errors are clustered at the industry level.

*** p<0.01,

** p<0.05,

* p<0.1.

### 5.3 Heterogeneity

This part examines whether the effects of EGTO are heterogeneous across different contexts, the results of which can help guide policy discussions and future studies on EGTO. First, we explore the heterogeneity regarding the ownership of enterprises. In China, state-owned enterprises (SOEs) are more obedient to government policies, hence are preferred by governments for achieving economic growth targets. Therefore, SOEs may be more susceptible to EGTO. To detect the heterogeneity, we first divide the enterprises in our sample into three groups based on ownership (i.e., SOEs, domestic private enterprises, and foreign enterprises), and then estimate the effect of EGTO for each group. Columns 1–3 in Table 6 display the results. In column 1 of panels A, B and C, we show that EGTO has a significantly stronger positive effect on the output of domestic private enterprises in high-polluting industries. In column 2 of each panel, we find that the effect of EGTO on domestic private enterprises in high-polluting industries is positive and statistically significant as well, but smaller in magnitude. However, there is no significantly stronger effect on foreign enterprises in water-polluting and both-polluting industries, while the effect on foreign enterprises in air-polluting industries remains significant (cols. 3), arguably because the production plans of foreign enterprises are constrained by their overseas parent companies who are insusceptible to the EGTO of Chinese governments.

**Table 6:** Heterogeneous effect of EGTO by ownership and scale.

	SOEs	Demestic private	Foreign	Large	Small
	(1)	(2)	(3)	(4)	(5)
***Panel A*. *Air-polluting industry***					
EGTO *dirty	0.069***	0.045**	0.043**	0.071***	0.042**
	(0.016)	(0.020)	(0.023)	(0.020)	(0.017)
Industry-year FE	Y	Y	Y	Y	Y
City-year FE	Y	Y	Y	Y	Y
City-industry FE	Y	Y	Y	Y	Y
Observations	4340	4340	4340	4340	4340
R-squared	0.927	0.914	0.874	0.913	0.887
***Panel B*. *Water-polluting industry***					
EGTO *dirty	0.059***	0.034*	0.020	0.052***	0.034**
	(0.020)	(0.018)	(0.022)	(0.017)	(0.015)
Industry-year FE	Y	Y	Y	Y	Y
City-year FE	Y	Y	Y	Y	Y
City-industry FE	Y	Y	Y	Y	Y
Observations	4340	4340	4340	4340	4340
R-squared	0.899	0.912	0.884	0.900	0.881
***Panel C*. *Both***					
EGTO *dirty	0.062***	0.048**	0.030	0.064***	0.043**
	(0.020)	(0.019)	(0.027)	(0.021)	(0.018)
Industry-year FE	Y	Y	Y	Y	Y
City-year FE	Y	Y	Y	Y	Y
City-industry FE	Y	Y	Y	Y	Y
Observations	4340	4340	4340	4340	4340
R-squared	0.939	0.883	0.884	0.923	0.891

Notes: Standard errors are clustered at the industry level.

*** p<0.01,

** p<0.05,

* p<0.1.

Second, we investigate the heterogeneous effect of EGTO on large and small companies. We define “large” and “small” by company’s annual sales. Specifically, large companies are those whose annual sales are higher than the median of our sample; Small companies are those with below-median annual sales. Columns 4 and 5 present the results. As shown, EGTO has a larger impact on the polluting activities of large companies in comparison with small companies.

Finally, we explore the heterogeneity in the effect of EGTO regarding the different announcement times of economic growth targets. Local officials refer to the targets of higher-level governments when setting economic growth targets. If local officials set economic growth targets before their superior governments, they are more likely to set targets based on local development conditions. If the growth target of prefecture-level cities postdates the provincial target, prefecture-level cities may refer to the superior target, and tend to set higher targets for promotions. Results in the panel A of Table 7 confirm our conjecture, for cities announcing their targets after the provincial target, the impact of EGTO on polluting activities is larger. This result shows that when the growth targets of local governments are announced after the date when their superior governments announce the growth target, which implies a larger pressure on the promotion, EGTO has a larger impact on polluting activities.

**Table 7:** Heterogeneous effect of EGTO by target’s announcement time and resource dependence.

	Dependent variables: industrial output					
	**Air-polluting industry**		**Water-polluting industry**		**Both**	
***Panel A*. *Announcement time***						
	**After**	**Before**	**After**	**Before**	**After**	**Before**
	**(1)**	**(2)**	**(3)**	**(4)**	**(5)**	**(6)**
EGTO *dirty	0.093***	0.063***	0.067***	0.043**	0.081***	0.057***
	(0.024)	(0.020)	(0.021)	(0.019)	(0.022)	(0.020)
Industry-year FE	Y	Y	Y	Y	Y	Y
City-year FE	Y	Y	Y	Y	Y	Y
City-industry FE	Y	Y	Y	Y	Y	Y
Observations	1024	3316	1024	3316	1024	3316
R-squared	0.867	0.924	0.928	0.836	0.877	0.949
***Panel B*. *Resource dependence***						
	**Air-polluting industry**		**Water-polluting industry**		**Both**	
	Resource-based cities	Non resource-based cities	Resource-based cities	Non resource-based cities	Resource-based cities	Non resource-based cities
EGTO *dirty	0.084***	0.057**	0.092***	0.033*	0.088***	0.043*
	(0.028)	(0.027)	(0.025)	(0.017)	(0.023)	(0.022)
Industry-year FE	Y	Y	Y	Y	Y	Y
City-year FE	Y	Y	Y	Y	Y	Y
City-industry FE	Y	Y	Y	Y	Y	Y
Observations	1808	2532	1808	2532	1808	2532
R-squared	0.892	0.901	0.889	0.913	0.897	0.900

Notes: Standard errors are clustered at the industry level.

*** p<0.01,

** p<0.05,

* p<0.1.

China’s resource-based cities feature a relatively simple industrial structure. These industries largely rely on natural resources and thus are high-polluting. Therefore, if the officials of these cities want to achieve the overweight of economic growth target, they have to pay more attention to the high-polluting industries than the officials of other cities. That is, EGTO will have a larger impact on the polluting-activities of resource-based cities than that of non resource-based cities. To detect the heterogeneity, we first divide the sample cities into resource-based cities and non resource-based cities according to the “National Sustainable Development Plan for Resource-Based Cities” released by the Chinese State Council, and then do the baseline regression for each sample. Panel B of Table 7 shows the results. As expected, the coefficients of interaction terms for resource-based cities are statistically larger than these for non resource-based cities for all specifications, which means that EGTO induces more polluting activities for resource-based cities than for non resource-based cities.

## 6. Mechanisms

We probe the mechanisms underlying the effects of EGTO on polluting activities in this section. More specifically, how did EGTO induce more high-polluting activities? Many studies find that in order to boost GDP growth, local governments turn a blind eye to the pollution generated in the process of production activities, even shielding high-polluting firms from punishment [51]. Since environmental regulation can improve environmental quality but comes at the cost of jobs, productivity, or other undesirable economic effects [52, 53], if the economic growth target is high, local governments will pay much attention to GDP regardless of the environmental pollution. Therefore, we infer that EGTO promotes polluting activities by weakening environmental regulation. To formally test the hypothesis, we first, following a study [15], use the frequency of words as to environmental protection in the government work report to measure the intensity of environmental regulation of a city, and employ the following equation:

$$y_{ict}=\beta_{0}+\beta_{1}{\text{EGTO}}_{ct}*dirty_{i}*\text{envir}\_{\text{r}}_{\text{ct}}+{\text{EGTO}}_{ct}*dirty_{i}+\varphi_{it}+\tau_{ic}+\lambda_{ct}+\epsilon_{it}$$ (3)

where envir_r_ct_ refers to the intensity of the environmental regulation of city c in year t. Results are presented in Table 8. The coefficients of the triple-interaction terms are significantly negative in all specifications, which means that the more environmental regulations, the weaker impact of EGTO on the relative output of high-polluting industries. Hence, environmental deregulation is the mechanism through which EGTO increases polluting activities.

**Table 8:** Effect of EGTO with different intensity of environmental regulation.

	Dependent variables: industrial output		
	Air-polluting industry	Water-polluting industry	Both
	(1)	(2)	(3)
EGTO *dirty*envir_r	-0.076**	-0.123***	-0.097**
	(0.038)	(0.042)	(0.039)
EGTO *dirty	0.023	0.084**	0.046*
	(0.052)	(0.037)	(0.029)
Dirty*envir_r	-1.746	-0.451	-0.356
	(0.901)	(0.553)	(0.112)
Industry-year FE	Y	Y	Y
City-year FE	Y	Y	Y
City-industry FE	Y	Y	Y
Observations	4340	4340	4340
R-squared	0.916	0.902	0.893

Notes: Standard errors are clustered at the industry level.

*** p<0.01,

** p<0.05,

* p<0.1.

## 7. Further analysis

To cope with the downward pressure on the economy induced by the 2008 global financial crisis, the Chinese government adopted various measures to spur economic growth. For example, in late 2008, China announced a 4 trillion-yuan ($586 billion) stimulus plan to boost domestic demand. Although the external economic environment was very bad, China still set the 2009 GDP growth target at 8% which is the same as that of the last five years. On the whole, developing the economy was of particular importance during this period, which implies that the government’s tolerance and preference for productive high-polluting industries may be enhanced after the crisis. Therefore, a natural guess is that the impact of EGTO on polluting activities will be larger after 2008. To explore the time variation in the effect of EGTO, we employ the following empirical model:

$$y_{ict}=\beta_{0}+\beta_{1}{\text{EGTO}}_{ct}*dirty_{i}*post2008+{\text{EGTO}}_{ct}*dirty_{i}+\varphi_{it}+\tau_{ic}+\lambda_{ct}+\epsilon_{it}$$ (4)

where *post*2008 is a dummy variable, which equals 1 if t>2008 and 0 otherwise. The coefficient of the triple interaction term, *β*_1_, is of primary interest. A positive coefficient, *β*_1_ > 0, indicates that the effect of EGTO on polluting activities increases after 2008.

Table 9 shows the expected results. The coefficients of the triple interaction terms are positive and significant in all specifications, which suggests that the larger positive effect of EGTO on the output of high-polluting industries enhanced after 2008, in other words, EGTO induced more polluting activities after the financial crisis.

**Table 9:** Effect of the 2008 global financial crisis on the impact of EGTO.

	Dependent variables: industrial output		
	Air-polluting industry	Water-polluting industry	Both
	(1)	(2)	(3)
EGTO *dirty*post2008	0.049***	0.021*	0.039***
	(0.013)	(0.012)	(0.012)
EGTO *dirty	0.036***	0.024**	0.038**
	(0.012)	(0.010)	(0.015)
Industry-year FE	Y	Y	Y
City-year FE	Y	Y	Y
City-industry FE	Y	Y	Y
Observations	3280	3280	3280
R-squared	0.939	0.865	0.898

Notes: Standard errors are clustered at the industry level.

*** p<0.01,

** p<0.05,

* p<0.1.

We restrict our sample period to 2006–2012 so as to exclude the impact of the establishment of the air pollution monitoring system in 2013.

## 8. Conclusion

Using data from the Annual Survey of Industrial Firms and the economic growth targets of provinces and prefecture-level cities from 2006 to 2014, this paper finds that the economic growth target overweight (EGTO) has a positive impact on the output of high-polluting industries relative to low-polluting industries, which demonstrates that EGTO can induce more polluting activities. This result is robust to alternative measures of EGTO and addressing endogeneity. We also find evidence that EGTO promotes polluting activities by relaxing environmental regulations of polluting activities in high-polluting industries. Finally, we document an increase in the effect of EGTO after the 2008 global economic crisis.

Although official promotion evaluation based on economic performance has played a vital role in China’s phenomenal economic growth in the past 40 years, our results show that it also brings a negative impact on the environment. For this reason, China should make the growth-target official evaluation system more environmentally friendly, such as adding more performance indicators related to the environmental quality. Indeed, this is exactly what is happening now in China. For instance, in 2013, the Central Committee of the Communist Party approved the “Decision of the CCCPC on Some Major Issues Concerning Comprehensively Deepening the Reform”, in which the weight of other evaluation indicators such as resource consumption, environmental damage, and ecological benefits has been increased. Thus, an intriguing line for future research is to examine whether the updated official evaluation system improves China’s environment, or brings costs to the economy. Finally, it is worth noting that the latest year when the data of the Annual Survey of Industrial Firms is publicly available is 2014. Therefore, the findings of this paper may not support current operations of high-polluting industries.

## Supporting information

S1 Appendix Pollution intensity index of different industries. (DOCX)

S2 Appendix List of high-polluting industries for different pollutants. (DOCX)
