# Supplementary material for: Does the economic growth target overweight induce more polluting activities? Evidence from China
Source: PLoS One. 2023 Mar 7;18(3):e0282675. doi: 10.1371/journal.pone.0282675 (PMC9990941; doi:10.1371/journal.pone.0282675)
Supplement: S1 Appendix — (DOCX) [file pone.0282675.s001.docx]

**S1 Appendix. Pollution intensity index of different industries.**

| **Adjusted Industrial Code** | **Industry** | **PII** | | |
| --- | --- | --- | --- | --- |
|  |  | **wastewater** | **sulfur dioxide** | **smoke** |
| 06 | Mining and Washing of Coal | 1.908 | 1.005 | 0.379 |
| 07 | Extraction of Petroleum and Natural Gas | 0.179 | 0.044 | 0.063 |
| 08 | Mining and Processing of Ferrous Metal Ores | 1.891 | 1.395 | 0.635 |
| 09 | Mining and Processing of Non-Ferrous Metal Ores | 4.748 | 0.573 | 1.461 |
| 10 | Mining and Processing of Nonmetal Ores | 1.507 | 2.578 | 1.097 |
| 12 | Mining of Other Ores | 23.587 | 35.331 | 6.298 |
| 13 | Processing of Foods | 3.100 | 0.601 | 0.461 |
| 14 | Manufacture of Foods | 2.550 | 0.563 | 0.705 |
| 15 | Manufacture of Beverages | 3.558 | 0.717 | 0.704 |
| 16 | Manufacture of Tobacco | 0.098 | 0.044 | 0.052 |
| 17 | Manufacture of Textile | 4.990 | 0.487 | 0.667 |
| 18 | Manufacture of Apparel, Footwear & Caps | 0.673 | 0.065 | 0.075 |
| 19 | Manufacture of Leather, Fur, & Feather | 1.733 | 0.138 | 0.147 |
| 20 | Processing  of Timber,  Manufacture of Wood, Bamboo, Rattan, Palm & Straw Products | 0.491 | 1.417 | 0.445 |
| 21 | Manufacture of Furniture | 0.244 | 0.204 | 0.049 |
| 22 | Manufacture of Paper & Paper Products | 23.186 | 2.324 | 3.239 |
| 23 | Printing, Reproduction of Recording Media | 0.232 | 0.045 | 0.050 |
| 24 | Manufacture of Articles For Culture, Education & Sport Activities | 0.213 | 0.065 | 0.025 |
| 25 | Processing of Petroleum, Coking, &Fuel | 2.901 | 3.422 | 3.109 |
| 26 | Manufacture of Raw Chemical Materials | 4.545 | 1.629 | 1.831 |
| 27 | Manufacture of Medicines | 2.041 | 0.361 | 0.396 |
| 28 | Manufacture of Chemical Fibers | 6.658 | 0.876 | 1.987 |
| 29 | Manufacture of Rubber | 0.844 | 0.700 | 0.616 |
| 30 | Manufacture of Plastics | 0.374 | 39.171 | 1.874 |
| 31 | Manufacture of Non-metallic Mineral goods | 0.998 | 6.078 | 4.215 |
| 32 | Smelting & Pressing of Ferrous Metals | 1.432 | 0.606 | 2.122 |
| 33 | Smelting & Pressing of Non-ferrous Metals | 0.782 | 0.197 | 1.733 |
| 34 | Manufacture of Metal Products | 0.897 | 0.368 | 0.153 |
| 35 | Manufacture of General Purpose Machinery | 0.235 | 0.081 | 0.096 |
| 36 | Manufacture of Special Purpose Machinery | 0.357 | 0.320 | 0.098 |
| 37 | Manufacture of Transport Equipment | 0.350 | 0.021 | 0.059 |
| 39 | Electrical machinery & equipment | 0.156 | 0.016 | 0.022 |
| 40 | Manufacture of Communication Equipment, Computers and Other Electronic Equipment | 0.391 | 0.007 | 0.015 |
| 41 | Manufacture of Measuring Instruments and Machinery for Cultural Activity and Office Work | 0.478 | 0.134 | 0.036 |
| 42 | Manufacture of Artwork and Other Manufacturing | 0.004 | 0.001 | 0.001 |
| 43 | Recycling and Disposal of Waste | 0.006 | 2.921 | 0.001 |
| 44 | Production and Supply of Electric Power and Heat Power | 2.163 | 0.038 | 15.017 |
| 45 | Production and Supply of Gas | 0.875 | 0.039 | 0.968 |
| 46 | Production and Supply of water | 6.274 | 0.106 | 0.074 |
