# Supplementary material for: Does the economic growth target overweight induce more polluting activities? Evidence from China
Source: PLoS One. 2023 Mar 7;18(3):e0282675. doi: 10.1371/journal.pone.0282675 (PMC9990941; doi:10.1371/journal.pone.0282675)
Supplement: S2 Appendix — (DOCX) [file pone.0282675.s002.docx]

**S2 Appendix. List of high-polluting industries for different pollutants.**

| **Water pollution industry** | **Smoke pollution industry** | **SO2 pollution industry** |
| --- | --- | --- |
| 13. Agricultural food processing industry | 20. Wood processing and wood, bamboo, rattan, palm and grass products industry | 22. Paper and Paper Products industry |
| 14. Food manufacturing | 22. Paper and Paper Products industry | 25. Petroleum processing, coking and nuclear fuel processing industry |
| 15. Beverage manufacturing | 25. Petroleum processing, coking and nuclear fuel processing industry | 26. Chemical materials and products industry |
| 17. Textile industry | 26. Chemical materials and products industry | 28. Chemical fiber manufacturing |
| 19. Leather, fur, feather (velvet) and their products industry | 30. Plastic products industry | 30. Plastic products industry |
| 22. Papermaking and paper products industry | 31. Non-metallic mineral products industry | 31. Non-metallic mineral products industry |
| 25. Petroleum processing, coking and nuclear fuel processing industry | 43. Waste resources and waste material recycling and processing industry | 32. Ferrous metal smelting and rolling  Processing industry |
| 26. Chemical materials and products industry |  | 33. Nonferrous metal smelting and rolling processing industry |
| 27. Pharmaceutical manufacturing |  |  |
| 28. Chemical fiber manufacturing |  |  |
| 32. Ferrous metal smelting and rolling processing industry |  |  |

Note: The two-digit industry category code comes from the “2002 Industrial Enterprise Industry Classification and Code”.
